# Production and Use of *Hymenolepis diminuta* Cysticercoids as Anti-Inflammatory Therapeutics

**DOI:** 10.3390/jcm6100098

**Published:** 2017-10-24

**Authors:** Kendra Smyth, Claire Morton, Amanda Mathew, Sahil Karuturi, Cliff Haley, Min Zhang, Zoie E. Holzknecht, Chelsea Swanson, Shu S. Lin, William Parker

**Affiliations:** 1 University Program in Ecology, Duke University, Durham, NC 27708, USA; kendra.smyth@gmail.com; 2 Department of Surgery, Duke University Medical Center, Durham, NC 27710, USA; claire.morton@duke.edu (C.M.); amanda.mathew@duke.edu (A.M.); sahilk2000@gmail.com (S.K.); cliffhaley222@gmail.com (C.H.); Min.zhang39@duke.edu (M.Z.); zoie.holzknecht@duke.edu (Z.E.H.); shu.lin@duke.edu (S.S.L.); 3 The Duke Brain Imaging & Analysis Center, Duke University Medical Center, Durham, NC 27710, USA; chelsea.swanson@duke.edu; 4 Mental Illness Research Education and Clinical Center for Post Deployment Mental Health, Durham VA Medical Center, Durham, NC 27710, USA; 5 Department of Immunology, Duke University Medical Center, Durham, NC 27710, USA; 6 Department of Pathology, Duke University Medical Center, Durham, NC 27710, USA

**Keywords:** helminthic therapy, helminth, biological therapeutic, inflammation, anti-inflammatory

## Abstract

Helminthic therapy has shown considerable promise as a means of alleviating some inflammatory diseases that have proven resistant to pharmaceutical intervention. However, research in the field has been limited by a lack of availability to clinician scientists of a helminth that is relatively benign, non-communicable, affordable, and effectively treats disease. Previous socio-medical studies have found that some individuals self-treating with helminths to alleviate various diseases are using the rat tapeworm (cysticercoid developmental stage of *Hymenolepis diminuta*; HDC). In this study, we describe the production and use of HDCs in a manner that is based on reports from individuals self-treating with helminths, individuals producing helminths for self-treatment, and physicians monitoring patients that are self-treating. The helminth may fit the criteria needed by clinical scientists for clinical trials, and the methodology is apparently feasible for any medical center to reproduce. It is hoped that future clinical trials using this organism may shed light on the potential for helminthic therapy to alleviate inflammatory diseases. Further, it is hoped that studies with HDCs may provide a stepping stone toward population-wide restoration of the biota of the human body, potentially reversing the inflammatory consequences of biota depletion that currently affect Western society.

## 1. Introduction

Helminthic therapy, the use of helminths to treat disease, offers the best hope of decreasing inflammation via immunomodulation rather than immunosuppression [1,2,3], and probably also improves mucosal barrier function [4]. The scientific foundation behind this hope is based on the long co-evolutionary history of helminths with their vertebrate hosts [5]. Forces driving that coevolution include advantages to both host and helminth in minimizing the impact of helminth colonization on host fitness [6,7]. This evolutionary process has resulted in the existence of helminths, which are benign under conditions of adequate nutrition, but yet modulate host immune function in a manner that decreases inflammation without impairing immune function [1,7,8].

The idea of using helminths to treat disease is relatively new: The first report of helminths alleviating an incurable condition was published by Turton in 1976, who found that self-treatment with hookworms relieved his seasonal allergies [9]. However, Turton’s approach was not useful on a broader scale due to substantial adverse side effects, and it was not until 2005 that the first clinical trials were published showing that apparently benign helminths could alleviate inflammatory bowel disease in humans [10,11]. Those studies were followed closely by work showing that accidental colonization with helminths halts the progression of the most common form of multiple sclerosis [12,13]. 

The utility of helminths to treat autoimmune disease is well documented in laboratory animal models [7], and the mechanisms underlying helminth-derived immunomodulation are being elucidated [14,15]. Work with experimental models also suggests that helminths may be useful in preventing or even treating some allergic conditions [16,17]. More recently, work in laboratory rats has shown that colonization with helminths can prevent inflammation associated neuropsychiatric dysfunction [18]. It is anticipated that the beneficial effects of helminths on neuropsychiatric conditions are mediated by reduction of the inflammation that is associated with those conditions [18,19].

Literally dozens of helminths exist that might be considered as candidates for use in helminthic therapy [20]. Yet, clinical trials conducted so far have been limited to either the porcine whipworm or the human hookworm. The porcine whipworm, which had shown great promise in initial studies [10,11,21], was hampered by very high production costs and possible problems with production methodology [22]. Some studies have followed in Turton’s footsteps and have used the human hookworm [23,24,25,26], but these studies have been limited to doses and time frames that may be inadequate for treatment with that organism [22]. Indeed, given the known potential for the human hookworm to cause gastrointestinal distress, and in some cases fatigue [27,28], it is understandable that investigators have approached the use of this organism with caution. In addition, the communicability of the human hookworm understandably dampens enthusiasm among regulators and public health officials for widespread therapeutic use of the organisms. With these considerations in mind, it seems both timely and helpful to examine the potential of helminths other than the porcine whipworm and the human hookworm for therapeutic use. Indeed, the lack of availability of helminths is considered to be a major limitation in the ongoing effort to utilize helminths for therapeutic purposes [29]. 

Flowers and Hopkins first pointed out that many individuals are “self-treating” with helminths, and that these individuals could provide valuable information regarding how helminths might be used successfully by clinicians for helminthic therapy [30]. The fundamental proposal was to evaluate the experience of self-treaters and determine which helminths might be useful, and which diseases might be effectively treated. This information would then be used as a basis for rigorous clinical studies. We have pursued this idea, systematically compiling anecdotes described by self-treaters in social media, books, videos, and other sources. In addition, we performed a detailed socio-medical study using (a) a survey-based approach that was able to capture outcomes free of survivor bias; (b) interviews with helminth producers and suppliers that had combined decades of commercial and non-commercial experience; and (c) interviews with physicians who have substantial experience with hundreds of patients that were/are self-treating [22,31]. Interviews with physicians corroborated other lines of evidence collected in the socio-medical study, lending confidence to the conclusions. Based on the study design and the results, it was concluded that the generally positive effects of helminthic therapy observed in the study were probably real and not primarily due to artifacts such as survivor bias, expectations of the participants, and normalization to the mean [22,31]. Based on that ongoing work, which now includes more than 1000 self-treatment cases, we hypothesize that the rat tapeworm, *Hymenolepis diminuta*, will be an effective organism for helminthic therapy. Supporting this view, the organisms are protective of immune function without adverse side effects in laboratory animals [1,18,32,33,34]. Based on established knowledge regarding rat tapeworms [33] and socio-medical studies [22,31], the rat tapeworm apparently has the following profile:

(a) Production is relatively straight-forward. Although producing effective organisms requires particular conditions, those conditions can be easily reproduced at any medical center with laboratory animal resources. The primary advantages of this organism in regards to production are that (1) the therapeutic life stage of the rat tapeworm is raised in grain beetles, which are non-toxic to humans, and (2) that the production and use of the organisms are not impeded by intellectual property rights.
(b) The risk of adverse side effects is apparently low.
(c) The organisms do not leave the lumen of the gut, in contrast to other organisms (whipworms and hookworms) that have been used for helminthic therapy.
(d) The organisms generally do not survive long in humans and are not communicable via human-to-human transmission.
(e) The organisms appear to be generally effective in the treatment of autoimmune conditions, neuropsychiatric disorders, inflammatory conditions of the bowel, and with some caveats, allergic conditions.

In this study, we describe our experience with the production of rat tapeworms in a manner that is based on protocols described to us by four individuals currently producing and supplying helminths for therapeutic use. The socio-medical-based protocol includes some modifications to accommodate laboratory conditions. We also describe our experience with purification of the therapeutic life stage of the helminth, *Hymenolepis diminuta* cysticercoids (HDCs). Such purification may be helpful in obtaining regulatory approval for clinical use of the organisms in the US. In addition, based on the collective observations, mostly made by physicians, of over 700 individuals self-treating with the organisms, we describe the treatment protocols for using HDCs, including dosage, frequency of exposure, and methods for optimization of dosage. Finally, the reported effects of self-treatment with the rat tapeworm, both positive and negative, are described. 

It is hoped that this report will provide a resource for investigators wishing to conduct clinical trials probing the potential for helminthic therapy to provide an effective and relatively risk-free method of treating a wide range of inflammatory diseases.

## 2. Materials and Methods

### 2.1. Socio-Medical Studies

Socio-medical studies were conducted, as previously described [22,31], with approval from the Duke University Institutional Review Board (approval number Pro00045035). Consent was waived for the evaluation of publicly available information, and the requirement to obtain a signed consent for individuals completing survey forms or participating in interviews was waived. At no time during the study was any protected health information gathered, ensuring the anonymity of the participants. Four approaches were utilized. Interviews with helminth providers were conducted, surveys from individuals self-treating with helminths were collected, publicly available information regarding self-treatment with helminths was compiled, and physicians treating patients who self-treated with helminths were interviewed. Of the 1066 experiences with helminthic therapy collected to date, most (707) utilized treatment with HDCs, and only those experiences utilizing HDCs were considered in this report. Most (*n* = 654; 92.5%) of the 707 experiences with self-treatment using HDCs were related to us by physicians monitoring patients that were self-treating. The remainders were either from surveys submitted by self-treaters (*n* = 44; 6.2%) or gleaned from publicly available information (*n* = 6; 0.85%). 

This report includes extensive details, including some pitfalls, in the production and therapeutic use of HDCs that have not been previously reported but which will be of potential use to investigators interested in conducting clinical studies with HDCs. The therapeutic effects and adverse side effects of self-treatment with HDCs have been described previously in two studies [22,31]. The first study [22], by Cheng et al., described the effects of HDCs in a population of adults who had completed a survey created by the authors. The second study [31], by Liu et al., described physicians’ observations of patients who were self-treating. This second study included 11-fold more individuals using HDCs than did the first study, but the results from these individuals were combined with that of individuals that had been self-treating with the porcine whipworm. In this report, the therapeutic effects of HDCs are presented independently of the effects of any other helminth, providing insight into the use of one particular helminth, rather than on the use of helminthic therapy in general. A total of 707 cases of self-treatment with HDCs (all data available to date) are considered in this report. 

### 2.2. Production of HDCs: Basis for Methodology

Three of the four suppliers that we interviewed provided full details regarding the production of their HDCs. All three suppliers used similar protocols, which had initially been developed by one of them, the “primary supplier”. The protocol from the primary supplier was used as a basis for the protocol described herein, although information from other suppliers was used to refine or clarify the protocol. One supplier used a production method that was independent of the other three and noted that some aspects of his/her production were proprietary. Another supplier noted that some aspects of his/her post-production purification procedures were proprietary, although he/she fully disclosed the production method. Nevertheless, all of the suppliers provided useful information upon which the protocol described herein was established. 

All of the suppliers cultivated HDCs from stocks that were either originally obtained directly from Carolina Biological Supply or from another source who had originally obtained their organisms from Carolina Biological Supply. Two suppliers noted that HDCs from Carolina Biological Supply, after appropriate aging (four weeks from date of delivery) were effective as therapeutic agents. Thus, the purpose of protocol development for the suppliers was aimed at effective production of organisms that had the same benefits as those observed with HDCs obtained from Carolina Biological Supply. Yet because Carolina Biological Supply produces HDCs for educational but not therapeutic purposes, in this study we did not consider the company to be a producer or supplier of HDCs for self-treatment. Unfortunately, efforts to obtain their production protocol were not successful. Nevertheless, the production protocol suggested to customers by Carolina Biological Supply (for educational purposes only) was similar in many regards to the protocols used by three of the four suppliers. The primary supplier confirmed that he/she developed his/her production method by systematically modifying the protocol from Carolina Biological Supply, with the idea of improving efficiency and ease of production without compromising the beneficial effects of the organisms. 

### 2.3. Production of HDCs: Overall Approach

**Figure 1:**
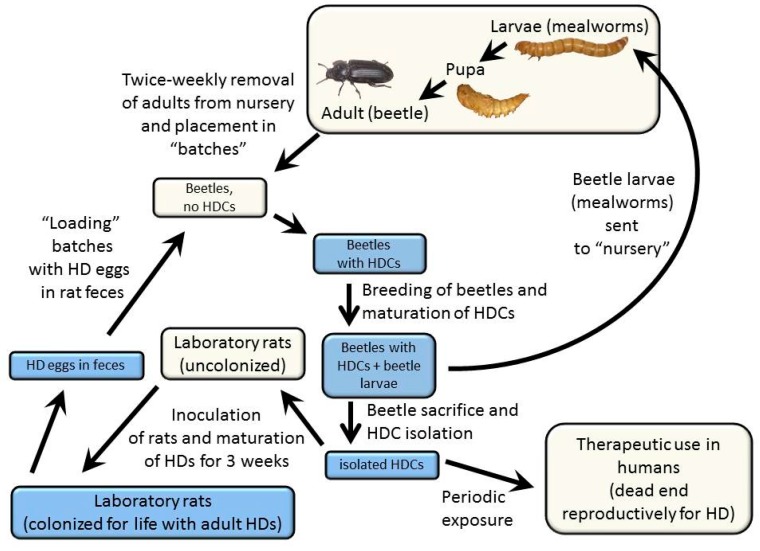
Schematic diagram showing maintenance of the rat tapeworm in laboratory rats (primary hosts) and grain beetles (secondary hosts) as described in the text. Blue boxes indicate steps that include live *Hymenolepis diminuta*, either eggs, *Hymenolepis diminuta* cysticercoids (HDCs), or adult tapeworms.

The overall approach to cultivating HDCs, the details of which are described below, is illustrated in Figure 1 and described as follows: The laboratory rat (*Rattus norvegicus*) serves as an effective primary host for the rat tapeworm, and the grain beetle (*Tenebrio molitor*) serves as an effective and practical secondary host. It is within the secondary host that the therapeutic stage of the organism, the HDC, develops. Rats are initially colonized with the rat tapeworm by feeding approximately five HDCs to each rat. This number of tapeworms ensures that each adult tapeworm can grow to maximum size and achieve a high rate of egg production. Colonization with more rat tapeworms results in a reduced size of each tapeworm, making it more difficult to distinguish the rat tapeworms from *Hymenolepis nana* (the dwarf tapeworm), a related but smaller species that can also colonize rats. After approximately 17 to 21 days following inoculation, tapeworms living in the small bowel of the rat begin to produce eggs. The rat’s fecal material, laden with tapeworm eggs, is then fed to grain beetles, which have been starved. After a period of feeding on the fecal material, the beetles are returned to their usual maintenance diet. The resulting HDCs live in the extra-intestinal space of the abdominal cavity of the grain beetles. Dissection of the beetles yields HDCs that are therapeutically active between approximately six weeks to five months after the beetles are inoculated. 

### 2.4. Maintenance of Rats and Inoculation with Tapeworms

Sprague Dawley rats (Harlan Sprague Dawley, Indianapolis, IN, USA) were maintained in AAALAC-approved barrier facilities at Duke University Medical Center in accordance with institutional guidelines. All animal care and procedures were approved by the institutional animal care and use committee at Duke University. Each rat was inoculated with 4–7 HDCs (originally obtained from Carolina Biological Supply, Burlington, NC, USA) suspended in 0.6% saline (ACS grade NaCl(s) from Mallinckrodt Chemicals, Phillipsburg, NJ, USA) delivered orally via a pipette. After 3 weeks, the rats excreted *Hymenolepis diminuta* eggs in their feces and continued to do so for four months or longer. Egg output was evaluated periodically (i.e., 21 days after inoculation and monthly thereafter) via fecal flotation, and when necessary, rats were re-inoculated with HDCs. 

The morphology of the HDCs was routinely examined (see Results) to confirm that the organisms were indeed *Hymenolepis diminuta*. However, because another species, *Hymenolepis nana*, closely resembles *Hymenolepis diminuta* at the cysticercoid stage, we confirmed that the species was *Hymenolepis diminuta* by examining adult organisms. During the course of this work, we evaluated more than 60 adult specimens from our stock and determined that all were *Hymenolepis diminuta* and not *Hymenolepis nana* based on size. (*Hymenolepis diminuta* are greater than 30 cm in length and *Hymenolepis nana* are less than 6 cm in length). Although other methods are available that can be used to distinguish *Hymenolepis diminuta* from *Hymenolepis nana*, an evaluation of adult size is the most convenient, not requiring specialized training (for evaluation of the morphology of the scolex (head) of the adult) or standards that are not commercially available (for evaluation of egg size).

### 2.5. Beetle Colony Maintenance

Colonies of grain beetles (*Tenebrio molitor*), originally obtained from Carolina Biological Supply, have been cultivated under controlled laboratory conditions at Duke University Medical Center since May of 2013. *T. molitor* larvae (mealworms) were housed in a “nursery” container where they grew for 6–10 weeks and pupated. (See details below). After pupae metamorphosed into beetles, beetles were transferred twice per week from the nursery into a secondary container for adults, or “batch” (see details below). Each batch was inoculated with HDCs, as described below. The nursery was replenished with mealworms, which hatched from eggs laid by adult beetles. When handing beetles (including adults, pupae, eggs, or mealworms), lab coats, gloves, and masks were worn to prevent contamination. In addition, care was taken to avoid exposure of technical staff to the allergens produced by the grain beetles. This was accomplished using personal protective equipment and by cultivating the beetles in a chemical fume hood. Further, individuals known to be sensitive to dust and mold were not involved in the production of the grain beetles.

Two nurseries were maintained, with each nursery being 31 cm × 27 cm (base) × 8 cm tall. A 20 cm × 6 cm opening was cut in each lid to allow for ventilation, and an aluminum wire screen (1.5 mm mesh size) was glued (MultiTemp gluesticks, Adhesive Technologies, Inc., Hampton, NH, USA) over the hole to prevent escape of the beetles. The nursery was maintained with approximately 60 g of dry oatmeal (Old Fashioned Quaker oats; Quaker Oats Company, Chicago, IL, USA) added once every two weeks to maintain a depth of oatmeal at approximately 1 cm throughout the nursery. In addition, 3–5 tablespoons of nutritional yeast seasoning (Bragg Live Food Products, Santa Barbara, CA, USA) was added to every cup of oatmeal. To provide the beetles with moisture, approximately 70 g of organic celery (minimum = 43.5 g; mean ± SD = 70.0 ± 13.3 when measured over a three month period) purchased from a local grocery store was added twice per week, and old celery was removed at the same time. Celery was washed and dried prior to use to remove gross debris. To reduce growth of mold in the nurseries, toothpicks (purchased at a local grocery store) were inserted into the celery to keep the celery from being submerged in the oat/yeast mixture. The toothpicks were inserted such that the surface of the celery just touched the surface of the oat/yeast mixture. 

Batches (adults harvested from the nursery at regular intervals as described above) were housed in an assortment of food-quality plastic containers modified for ventilation. The average surface area at the base of the batch containers was 189 cm^2^ (range 182–243 cm^2^) and the average height was 4.7 cm (range 3.3–6.0 cm). An opening for ventilation was cut in each lid that averaged 41% of the surface area of the base of the container (range 22 to 53%). A wire screen was glued over the holes in the lids as described above. In cases where less than 25 adult beetles were housed, “small batch containers” were used to conserve space. These food-quality containers had a surface area at the base of 55.6 cm^2^ and a height of 5.0 cm. Holes were cut in the lids of these containers that averaged 53% of the surface area at the base of the container (range 46 to 58%). The adult (batch) containers were provisioned with approximately 60 g of oatmeal, without nutritional yeast. Oatmeal was added to batch containers as needed, and all mealworms (offspring of the adults in the batch) were transferred from batch containers to nurseries when some of them within the batch began to pupate. As a source of water, organic celery was refreshed twice weekly (minimum = 25.0 g/batch; mean ± SD = 32.2 ± 9.6 g/batch when measured over a three month period). Each batch container also included a plastic dome approximately 7 cm × 7 cm (length × width) and about 1.4 cm tall (made from a section of a polypropylene drinking cup purchased at a local department store), which was used to separate the celery from the oats and also for environmental enrichment. 

Nurseries and batches of adults were stored together in larger containers or “incubators” (64 Quart Access Tote, 49.5 cm by 36.5 cm base, 38 cm tall, lid dimensions (largest dimensions) 55.9 cm by 44.7 cm. Rubbermaid, Winchester, VA, USA), modified to include ventilation holes. Each incubator was equipped with an Eva-Dry EDV-100 petite dehumidifier and a battery powered probe to monitor temperature and humidity. Incubators were stored inside a chemical fume hood and typically housed one nursery and up to eight batches. Beetles remained in the fume hood at all times, with one exception: One to two times per month, the nursery was removed from its incubator, taken to a large sink, and cleaned. For this cleaning procedure, the nursery contents were dumped into a sieve (1.65 mm mesh size), which retained the mealworms and pupae but allowed the droppings to pass through. The nursery container was rinsed and dried, and the mealworms and pupae were returned to the nursery and provided with fresh organic oats, celery, and nutritional yeast seasoning. 

### 2.6. Inoculation of Beetles with Hymenolepis Diminuta

Beetles that had matured to the adult stage within the previous three weeks were selected for inoculation and transferred into a new container (9 cm × 12 cm base, 6.5 cm tall) where they were maintained without food (oatmeal) and water (celery). After two days without food or water, beetles were fed fresh (i.e., collected in the morning and stored for no more than two days at room temperature) egg-laden rat fecal pellets. Beetles were fed such that pellets were in excess. None of the beetles were prevented from reaching fecal pellets due to overcrowding. Prior to being fed to the beetles, any fecal pellets that showed signs of dryness were re-hydrated by adding drops of distilled water to the pellets until they appeared moist. Excess water not absorbed by the fecal pellets was removed from the pellets before they were fed to the beetles. Beetles were fed fresh, moist pellets for two consecutive days, and then on the third day the beetles were transferred into a fresh “batch container” with oats and celery as described above. These inoculated beetles were considered a completed “batch”, and no additional beetles were added to the batch. After at least seven weeks, mature HDCs were harvested from the beetles and used as needed for experiments described below.

### 2.7. Harvest of HDCs from Grain Beetles

Almond Milk was used as a carrier solution for HDCs in order to prevent the HDCs from sticking to the container or pipette in which they were stored. Other forms of milk (bovine milk, rice milk, soy milk) are also sufficient for this purpose. Almond Milk was particularly suitable for our work since it is sterile when purchased and since it can be readily clarified in order to visualize HDCs. Almond Milk (Silk^®^ Original Unsweetened Almond Milk; WhiteWave Foods Company, Denver, CO, USA) was used as the solution for isolation of HDCs. The milk was used in the condition purchased from a local grocery store in some cases. In other cases, when it was necessary to visually observe the HDCs in solution, the Almond Milk was “clarified” to yield clarified Almond Milk (cAM). To clarify the Almond Milk, it was aliquoted at room temperature into 50 mL conical polypropylene tubes (Port City Diagnostics, Wilmington, NC, USA) and then frozen at −20 °C. The evening before an experiment, aliquots were removed from the freezer and thawed overnight at room temperature. The next morning, thawed aliquots were centrifuged for 10 min at 1313× *g*. After centrifugation, the supernatant (cAM) was removed using sterile technique and used that day. 

Each beetle was dissected on a 10-cm polystyrene petri dish (VWR International, Suwanee, GA, USA). The head and the thorax were removed with a swift motion using a clean scalpel. The legs, wings, and wing covers were removed using scissors, and the abdomen was placed wing-side up in a new, sterile petri dish containing approximately 750 uL of cAM. Using two pairs of forceps, the interior of the beetle abdomen was gently broken apart and scraped out into the cAM. The tip of a sterile Samco 231 disposable fine-tip transfer pipette (Port City Diagnostics, Willington, NC, USA) was used to gently suspend the contents of the beetle’s abdominal cavity, including the HDCs, in the milk. 

The petri dish was placed under a dissecting microscope and HDCs were identified (at 40× magnification) by characteristic morphology. Specifically, positive identification of an HDC required that each of the following morphological features be identified: (1) the overall shape, including the head and a tail; (2) the two “eye spots” in the head of the organism; and, (3) the gelatinous coating around the head. HDCs were harvested using a sterile Samco 231 disposable fine-tip transfer pipette, with care taken to avoid transferring any beetle remains. 

### 2.8. Separation of HDCs from Bacteria

**Figure 2:**
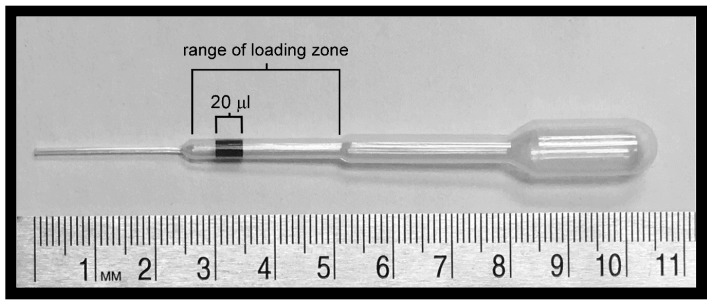
Loading of a Samco 231 disposable fine-tip pipette with HDCs. A 20 mL volume of opaque liquid has been loaded into this pipette. The numbers on the scale indicate centimeters.

To remove bacteria and remaining beetle parts from HDCs, HDCs were washed repeatedly or passed through a column. For the washing procedure, 10 HDCs were harvested from beetles (as described above) and transferred, using a sterilized Samco 231 disposable fine-tip transfer pipette, in approximately 20 uL, to 750 uL cAM in the center of a sterile petri dish. Because the transfer pipettes were hand loaded, an area representing approximately 20 uL was marked on the chamber of each pipette and used as a guide. Additionally, when transferring HDCs, they were retained in the 2nd chamber of the transfer pipette, marked “range of loading zone” in Figure 2, to prevent them from sticking or being trapped in junctions between chambers. Next, after being added to the 750 uL pool of cAM, HDCs were washed by gently swirling them in a circular motion approximately 20 times. At this point in the procedure, HDCs were either washed up to nine more times using the technique described above, or for purification using sedimentation, the HDCs were loaded onto a column.

To devise a column for separating HDCs from bacteria, the plug was removed from a 5-mL Kimble glass disposable serologic pipette (Port City Diagnostics, Wilmington, NC, USA), and a 5 cm segment of 1/4 inch outer diameter food-grade Norprene tubing (Cole-Parmer, Vernon Hills, IL, USA) was attached firmly onto the tip of the pipette. The column was placed in a Cardinal Health steam sterilization pouch (VWR International) and sterilized by steam under pressure in an autoclave. When ready for use, the column was held upright by a clamp attached to a ring stand. Prior to loading the column, the tubing at the bottom of the column was clamped shut to prevent liquid from flowing through the column. The column was then filled with 5.5 mL cAM with 0.6% saline added to increase the density of the liquid. Next, HDCs in 500 uL of cAM without added saline were loaded on top of the cAM with saline. Care was taken to ensure that HDCs, visible to the naked eye, did not stick to the column walls. A Scienceware three-way fluoroplastic stopcock (VWR International) was attached to a 10 mL syringe (BD Bioscience, San Jose, CA, USA) via a horizontal port and connected to the top of the column with 3/8 inch outer diameter PVC tubing (Cole-Parmer) via the vertical (middle) port. The column was sealed by rotating the dial on the stopcock from the horizontal to the vertical position. 

The HDCs were allowed to settle to the bottom of the column for 10 min, and then the clamp at the bottom of the column was removed. The contents of the column were collected by controlled expulsion from the column using the syringe. In this manner, aliquots of 500 uL each were collected in sterile 1.5 mL polypropylene microcentrifuge tubes (VWR International). HDCs were eluted in the first fraction (Fraction 1). Appropriate portions of fractions were spread on agar plates, previously made with with 27.8 g Miller LB broth (EMD Millipore, Billerica, MA, USA) and 16.7 g Difco granulated agar (BD, Franklin Lakes, NJ, USA) per liter of distilled water, and bacteria were quantified using the limiting dilution technique. Plates were incubated for 48-h at room temperature before colony-forming units (CFUs) were counted. 

### 2.9. Testing for Compliance with 21 CFR Part 11

Regulations in the United States (U.S.) (21 CFR Part 11) currently dictate that biological therapeutics administered orally must have concentrations of bacteria less than 100 CFUs/mL. To determine if our technique of washing was sufficient to achieve this standard, HDCs were washed 10 times as described in the Methods, and a total of 10 HDCs and 50 mL of Almond Milk were placed into a sterile 50 mL conical tube. Although it is anticipated that the dose of HDCs used for patients will be in the range of 0.5 HDCs/mL, a lower concentration (0.2 HDCs/mL) was submitted for analysis because (a) a 50 mL volume was required for analysis; and, (b) the intent was to test 10 HDCs at a time since that was the number isolated in a single series of washes. Samples containing 10 HDCs in 50 mL of liquid were stored at −20 °C for at least 24 h prior to shipping, and then shipped overnight in Styrofoam insulted boxes wrapped in bubble wrap with 5 to 7 refrigerant packs (equilibrated to −20 °C) per 10 samples of 50 mL each. Refrigerant packs were either 10 cm × 11.5 cm × 2 cm PolarPacks (Sonoco ThermoSafe, Arlington Heights, IL, USA) or 9 cm × 13 cm × 2.5 cm Cold Ice refrigerant gels (Cold Ice, Inc., Oakland, CA, USA). Samples packaged in this fashion were submitted to Diebel Laboratories (Lincolnwood, IL, USA) for the following tests: Aerobic Plate Count, Yeast ParmCo USP, Mold PharmCo USP, and *Escherichia coli* USP. 

## 3. Results

### 3.1. Production of HDCs

HDC production has been maintained at Duke University since May of 2013, with laboratory rats hosting adult tapeworms initially maintained in conventional laboratory housing (individually ventilated cages with 70 air changes per hour and reverse osmosis water). As of April of 2016, barrier housing (Helicobacter and Pasteurella free facility, sterilized cages and bedding, HEPA filtered cage change hoods, individually ventilated cages with 70 air changes per hour, reverse osmosis water) has been used to maintain rats hosting adult tapeworms. Since May of 2013, no other helminths have been found to be associated with the laboratory rats (primary hosts). 

The beetle production system, described in the Methods, resulted in the production of 37 beetles ±19.41 (range 9–99) twice per week in one nursery, and 43 ± 19.40 beetles (range 23–99) twice per week in the second nursery. There were no significant differences in either temperature or humidity in the two incubators as measured over a three-month period. In addition, the humidity in both incubators was not significantly different than that measured outside the incubators. The temperature in each incubator, on the other hand, was significantly higher than that measured outside of the incubators, averaging 2.13 °C (*p* = 0.001) and 2.04 °C (*p* = 0.004) higher inside as compared to outside of the two incubators. 

Microbial growth was evident in both incubators, and was reduced by (a) use of the dehumidifiers; (b) the use of celery as a water source rather than carrots or potatoes; (c) preventing celery from being submerged in the yeast/oat mixture; (d) bi-weekly changing of the celery; and (e) cleaning of the incubators as needed and regular cleaning of the nursery as described in the Methods. However, care was taken not to disinfect the incubators, with the idea of maintaining a steady-state ecosystem within the incubators. In addition to a wide range of yeast and bacteria, mites ranging in size from approximately 0.2 to 0.5 mm in body length were frequently associated with the adult beetles. 

**Figure 3:**
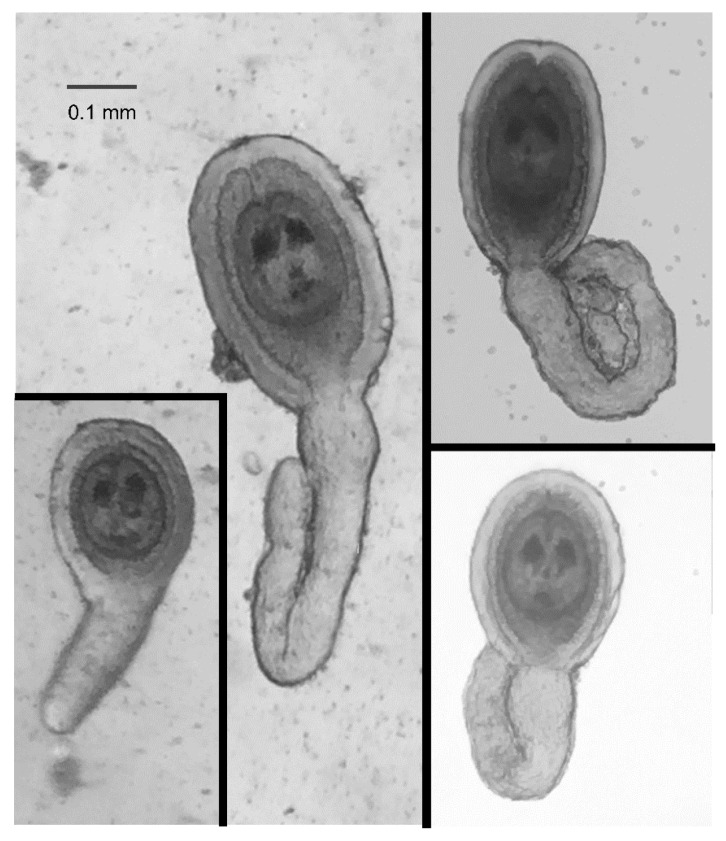
Typical HDCs. HDCs vary in size over roughly a 5-fold range, and have substantial morphological variation in their “tail”. Photographs shown were taken using iPhone 6 cameras and either an OMAX dissecting microscope with a iDu LabCam Microscope Adapter for the iPhone (**Left** images) or a Wilovert inverted microscope (**Right** images) by two coauthors (CM and ZEH) working independently.

HDCs were readily identified based on their general shape, a disk-shaped and relatively opaque “head” surrounded by a gelatinous coating and attached to a relatively transparent “tail” of varying lengths (Figure 3). This general shape, however, could be naively mistaken for materials from the beetle were it not for the two characteristic “eye spots” in the head of the HDC (Figure 3). The size of the head, including the thickness of the jelly coating varied by a factor of between 2 and 3, but the majority of the variation in the morphology of the HDCs was exhibited in the tail length. 

**Figure 4:**
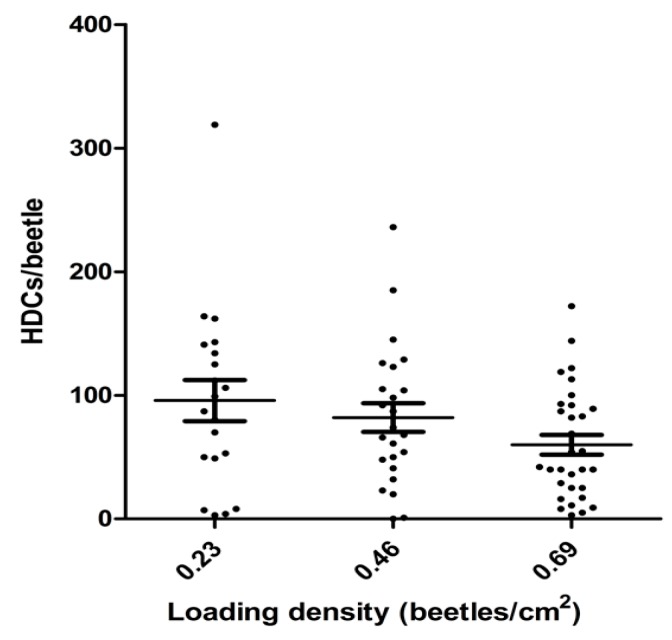
The effect of housing density on the efficiency of inoculation as measured by the number of HDCs obtained per beetle. The housing density during inoculation was varied, and after an appropriate incubation as described in the Methods, the inoculation efficiency was determined. Each data point represents the number of HDCs found in a single beetle. The loading density showed an inverse correlation with loading efficiency (m = −0.81 ± 0.36; *p* = 0.028).

The effect of beetle housing density during inoculation with *Hymenolepis diminuta* on the inoculation efficiency is shown in Figure 4. The housing density showed an inverse correlation with efficiency of inoculation (*p* = 0.028), but lowering the housing density over approximately a 3-fold range resulted in an increase of only about 30% in average inoculation efficiency as measured by the number of HDCs/beetle. Further, variation in the loading density at a given housing density was substantial (Figure 4), suggesting that housing density during loading is a relatively minor factor among others, which affect the efficiency of inoculation.

### 3.2. Pitfalls of Production that May Decrease Therapeutic Benefit

Some putative “pitfalls” with production were pointed out during interviews with one of the suppliers of HDCs used by individuals self-treating. These pitfalls were identified when attempts to enhance production methods were employed and the therapeutic benefits of the organisms were judged to be decreased or lost. Although the degree of certainty regarding the existence of at least some of these pitfalls is not absolute, they merit inclusion in this manuscript. Testing the effects of subtle changes in production protocols on the therapeutic benefits of helminths is likely to be extremely costly and impractical, so the “best guess” from individuals self-treating is probably the best option for starting clinical trials, even if that is only a guess. 

Of the four suppliers of HDCs interviewed, one was “primary” in that this supplier provided protocols, which were utilized by two of the other suppliers (see Methods). This “primary supplier” noted several pitfalls in production methods. First, attempts to improve the beetle production method by using complex nutrition found in chicken feed (one trial) or dog food (another trial) resulted in an improved production of grain beetles, but also in helminths that lacked therapeutic benefit. Thus, it was concluded that a simple diet consisting of oats was preferable, despite the fact that the production of grain beetles was less efficient than might be obtained otherwise.

A second pitfall noted by the primary supplier was that, following inoculation of beetles with tapeworm eggs, approximately 6 to 7 weeks was required before the resulting HDCs were therapeutically effective. It was noted that HDCs at three weeks sometimes looked mature based on observations under the microscope, but that these organisms nonetheless lacked effectiveness. In our own experience, we have noted that some HDCs can take as long as 10 to 12 weeks to mature. However, most mature much more quickly, within 5 weeks. A third pitfall noted by the primary supplier was that after approximately 5 months in the beetle, HDCs lost effectiveness. 

The primary supplier observed a loss of effectiveness when bacterial overgrowth occurred in stored HDC preparations, or when the HDCs were added to degassed (boiled and cooled) liquids. The supplier speculated that a lack of oxygen might result in the death of the HDCs and subsequent loss of therapeutic effectiveness. In addition, one supplier and two medical doctors indicated that commercially available HDCs, which had been cleaned and shipped were approximately 30 to 50% less effective than fresh HDCs. However, it was also suggested that this loss of activity could be compensated for by increased dosage.

The supplier who developed a proprietary HDC production protocol independent of the other three suppliers noted that storage at 4 °C could result in loss of the therapeutic effect of the organisms. That supplier suggested 10 °C as a better storage temperature. However, this conclusion was not corroborated by other suppliers, suggesting that appropriate storage conditions may be dependent on the method of preparation of the organisms. 

The primary supplier noted that washing or storing of the HDCs in saline solutions results in sticking of the HDCs to a variety of plastic or glass containers. Our own experience with the organisms confirmed this observation. Thus, Almond Milk (suggested by the primary supplier) or clarified Almond Milk were used in all experiments to ensure efficient transfer of HDCs from one container to the next. 

The primary supplier noted that HDCs may be less effective therapeutically if the number of HDCs in a given beetle is too high. However, the supplier noted that this idea had not been “tested carefully”, and that this view may be the result of a bias against the very high ratios of HDC/beetle that were obtained when more complex nutrition (chicken feed or dog food) was used for production (see above). The supplier noted that 70 HDC/beetle was “definitely good”, and that up to 120 HDC per beetle was “probably OK”, but that over 200 HDC/beetle “might not be good”. The supplier noted that this view is speculative, and that the ratio of HDC/beetle may be unimportant. 

### 3.3. Purification of HDCs

**Figure 5:**
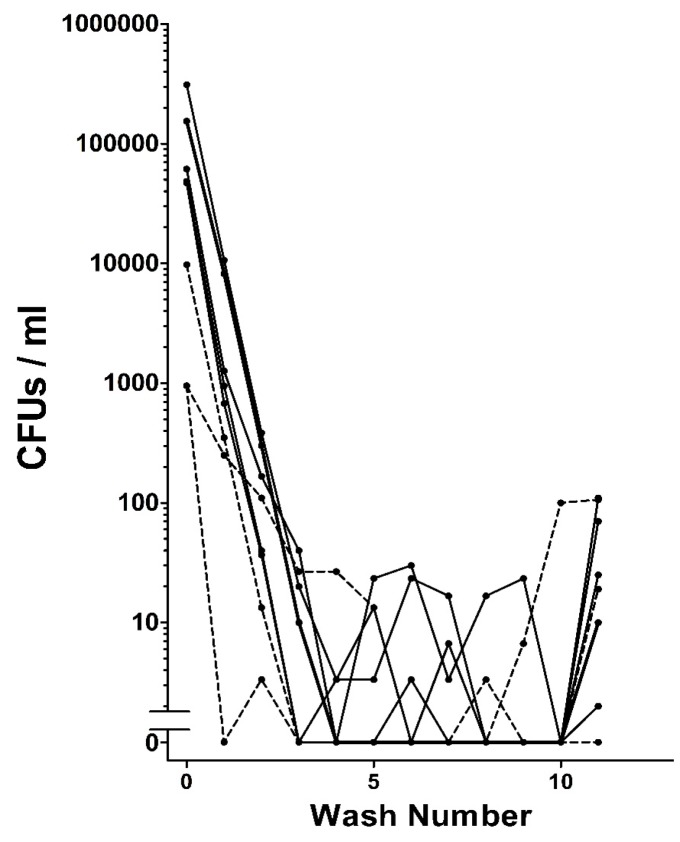
Removal of bacteria from HDCs by washing. The results from 8 trials with 10 HDCs being washed in each trial are shown. The final number in each trail is the number of bacteria associated with the HDCs after removal from wash 10. In 7 out of 8 trials, there were no bacteria observed in wash 10. However, in 7 out of 8 trials, there were bacteria associated with the HDCs.

When extracted from grain beetles, HDCs were found to be associated with bacteria. Repeated washing of the HDCs as described in the Methods removed the vast majority or even all of the bacteria that could be detected on agar plates (Figure 5). In some cases leaving washed HDCs on agar plates for several weeks yielded no microbial growth, suggesting that the HDCs do not have an intrinsic microbiome. 

Following 10 washes in clarified Almond Milk, freezing, and subsequent testing as described in the Methods, 20 out of 20 samples exceeded USP criteria. All of the samples contained <10 CFU/g for Aerobic Plate Count, <10 CFU/g Yeast and Mold PharmCo USP, and were negative for *Escherichia coli*. However, shipping the samples at 10 °C rather than shipping the samples frozen resulted in much higher bacterial counts (>1000 CFU/mL), despite low pre-shipping bacterial counts determined using in-house limited dilution methods. These observations might suggest that freezing is necessary in order to avoid rapid bacteria growth and obtain accurate assessments of microbial burden in the HDC preparations. It is important to note, however, that freezing of samples intended for use as therapeutic agents in humans is not recommended, as the HDCs may be killed or damaged. 

**Figure 6:**
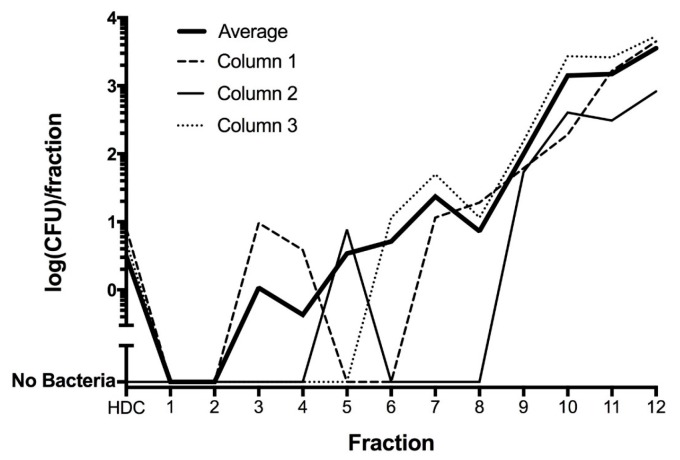
Purification of HDCs by sedimentation through a column. The number of bacteria decreased over the length of the column, such that no bacteria are present in the first and second fractions. Each fraction was approximately 0.5 mL in volume, except for the “HDC fraction”, which was taken from fraction 1 and contained the HDCs in a volume of 130 uL.

To evaluate whether bacteria associated with HDCs after washing could be removed by gentle mechanical means, HDCs were placed on a column and separated from bacteria using rapid sedimentation, as described in the Methods. These experiments confirmed that HDCs could be separated from all bacteria in some cases, but in other cases bacteria were apparently adherent to the HDCs (Figure 6).

### 3.4. Administration and Dosage of HDCs

**Figure 7:**
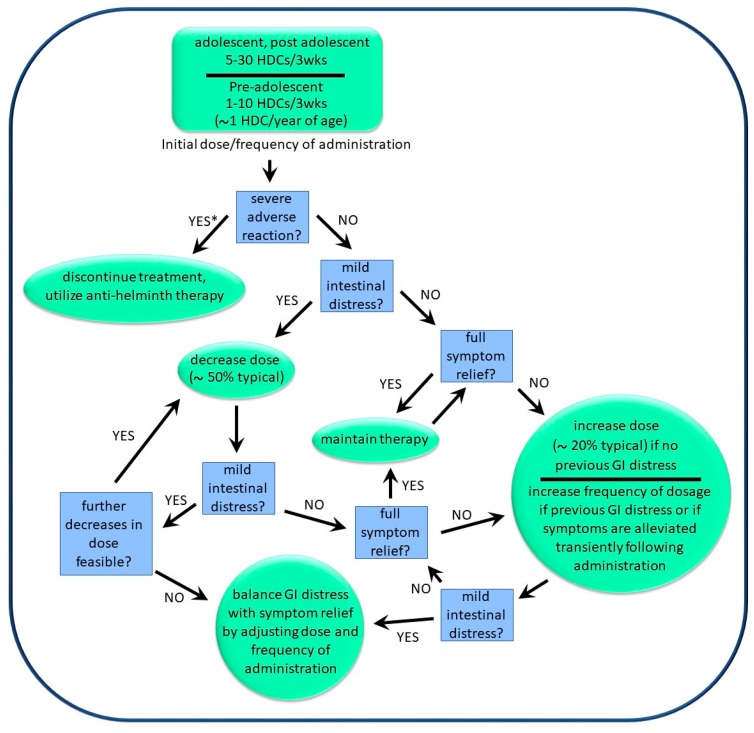
Decision tree for determination of optimal personal dosage and frequency of dosage of HDCs. * Substantial adverse reactions are rare and have thus far been limited to the pediatric population.

The dosage and frequency of administration of HDCs were described to us by six medical doctors supervising patients who were self-treating with the organisms. In addition, producers/suppliers of helminths also provided input in this regard. Information concerning dosage and frequency was generally supplied as “typical”, with minima and maxima sometimes given. The general approach used to “optimize dosage” for a given individual is shown in Figure 7. The approach was described by one helminth producer as being “akin to finding an optimal personal routine for physical exercise”, and involved starting at a “low dose” (see below), with gradual but steady increases in the dosage and/or frequency of dosage until either total symptom relief was obtained or mild and temporary diarrhea was experienced within 24 h of administration. The frequency of dosage was typically between 3 and 6 weeks, but could be as frequent as once per week. If transient diarrhea was observed following administration, then the dosage could be lowered while increasing the frequency of administration (Figure 7). Using this approach, the treatment regimens used by various patients ranged from 5 HDCs every 3 weeks to 100 HDCs per week. Physicians were unable to determine indicators that might be used to predict which patients required larger doses versus those that required smaller doses. However, pediatric patients generally used lower doses than did adults. 

The initial dose of HDCs used by adults typically ranged from 5 to 30 HDCs, and was typically administered every 3 to 6 weeks. Pediatric subjects typically started with 1 to 10 HDCs, again administered every 3 to 6 weeks. The dosage was then optimized as described above. Of note was an approximately 6 to 12 month “honeymoon period”, in which the effective dosage of HDCs was lower than the dosage needed for long-term maintenance. In general, the effective dosage during the first 6 months was gradually increased by roughly 2 to 3-fold for effective long-term maintenance (>6 years).

After optimization as described above, adult patients typically used treatment regimens for long term maintenance between 10 HDCs every 3 weeks to 100 HDCs per week, while pediatric individuals typically used treatment regimens between 3 HDCs every 3 weeks to 20 HDCs every three weeks. A few pediatric patients used weekly administration of HDCs, but this was a small minority (~1%). Weekly administration is generally more expensive than bi-weekly or monthly administration, so it is possible that financial considerations limited this option. 

All of the treatment regimens described above are for “fresh HDCs”, which are extracted from beetles and used without further purification. As previously described [22], commercially available HDCs that were cleaned and stored with antibiotics to retard bacterial growth during shipping had 30 to 50% less activity than did fresh HDCs. Thus, the effective dosage of commercially available HDCs was 50 to 100% greater than that of “fresh HDCs”.

The primary supplier (see Methods) noted several factors that might affect the therapeutic benefits of the helminths. First, avoidance of very hot liquids or high concentrations of alcohol immediately following the administration of HDCs was recommended. The supplier did not have any specific examples of circumstances when hot liquids or high concentrations had rendered a dose of HDCs ineffective, but believe that these conditions (heat and high concentrations of alcohol) should be avoided until the HDCs had transitioned from the stomach to the small bowel. However, the supplier did indicate that beer (alcohol content 5–7%) did not impair the therapeutic effect of HDCs, even when consumed concurrently with HDCs.

The primary HDC supplier also indicated that certain probiotics would interfere with “systemic effects” of the helminths. That is to say, certain probiotics would temporarily block effects observed on the skin, the sinuses, and the brain, but not in the gut. The supplier suggested that milk-based probiotics would decrease beneficial systemic effects of the HDCs, whereas probiotics that were not milk-based would have no effect. In particular, bifidobacteria and lactobacilli were noted as being problematic, whereas Ohhira’s^®^ probiotics and *Saccharomyces boulardii* were noted as being benign and, in some cases, helpful when taken with the HDCs. 

Finally, although no systematic evaluation of the interactions between helminths and pharmaceuticals has been performed, self-treaters apparently use HDCs in conjunction with standard medical care. Several physicians and suppliers of HDCs reported that the helminths work well in combination with standard pharmaceutical interventions, such as over-the-counter drugs used for seasonal allergies, prescription medications for inflammatory bowel disease, and prescription medications for neuropsychiatric disorders. 

### 3.5. Effects of HDC Use

The general effects of HDC use by self-treaters as compiled in our socio-medical study are summarized in Table 1. Based on physician reports, approximately 50% of individuals with inflammatory-related illness benefited from exposure to HDCs. However, approximately 95% of these reports were derived from self-treatment by the pediatric population, and most of these individuals had autism spectrum disorders with associated inflammation. Therefore, the available information is skewed to this population. A further caveat is that most individuals who discontinued therapy did not “optimize therapy”, as described above (Figure 7). Reasons for failure to optimize therapy included the financial costs and inconvenience in obtaining therapy. In addition, many individuals were apparently unaware that dose optimization can be helpful, apparently considering the therapy similar to a pharmaceutical with a set dosing regimen. Thus, in most cases when therapy showed now effect, it remains unknown whether the therapy itself was ineffective or whether the administration of the therapy was at fault. 

As might be expected, the reported effectiveness of HDCs depended on the purpose of the therapy. Conditions effectively treated included various anxiety disorders and other neuropsychiatric disorders, including migraine headaches, Tourette’s syndrome, chronic fatigue syndrome, and major depressive disorder. Autoimmune conditions, such as multiple sclerosis and inflammatory bowel disease, were apparently also treated effectively. Mixed results were obtained treating allergies and arthritis, and a very limited number of reports suggested that self-treatment with HDCs may treat lupus, Hashimotos’s disease, and Parkinson’s disease, but not Alzheimer’s disease. Some self-treaters noted an improvement or even a resolution of conditions which were not their reason for self-treatment. These included hemorrhoids (resolved), cardiac arrhythmias (resolved), and varicose veins (some improvement). 

Of the 707 total cases evaluated, several adverse effects of self-treatment were noted, all in the pediatric population. Most pediatric individuals with autism experienced a slight but transient increase in hyperactivity following self-treatment. However, this was not considered a reason to discontinue therapy. Rather, most individuals who discontinued therapy fell into the roughly 50% of people who did not experience substantial benefits and discontinued therapy for that reason. On the other hand, more troublesome adverse reactions were noted and included severe gastric pain associated with documented colonization (*n* = 3; 0.4%) and worsening of behavioral symptoms (*n* = 3; 0.4%). These more troublesome effects were relieved by treatment with anti-helminthic drugs and were cause for discontinuation of helminthic therapy. 

## 4. Discussion

Helminths have a largely unexplored potential to treat a wide range of inflammation-related diseases [3], and may prove very effective at treating some dread diseases that have proven extremely resistant to pharmaceutical based approaches. At the same time, based on sociomedical studies [22,31], as well as some clinical trials [10,35], some conditions are likely to prove resistant, and not all individuals will respond well. Supporting this view, studies in animal models demonstrate that, although helminths do help regulate immune function, they do not effectively treat all inflammatory diseases, and in some cases make certain pre-existing conditions worse, and they may not affect many diseases that are a “hard-wired” result of genetic mutation [36,37,38,39]. This report describes in detail the production and use of a potentially effective helminth for therapeutic use based on socio-medical data, and brings into focus the potential benefits and risks of that helminth, particularly in a subset of the pediatric population. The production of therapeutically effective HDCs apparently requires particular conditions, and deviation from working protocols can result in reduced therapeutic activity. Indeed, pitfalls in production of therapeutic helminths are probably not novel, as improper formulation of the porcine whipworm may have derailed efforts to use that organism in the clinical setting [31]. That being said, the protocol described herein is within the parameters that have been given to us by suppliers whose customers report physician-corroborated beneficial effects from the use of the helminths. The production of a therapeutically active HDC is apparently not technically challenging. The methodology to produce effective HDCs has been transferred successfully from supplier to supplier more than once, suggesting that widespread use of effective production methods may be readily achieved. 

Current US Food and Drug Administration (FDA) guidelines for oral biological therapeutics require that the number of CFUs be less than 100/mL in the final preparation. However, HDCs in the “raw” form, without removal of the associated bacteria, can contain greater than 1000-fold more bacteria than are permitted under the current regulations. Using the repeated washing approach described in the Methods, we were successful in removing bacteria in a manner that met current guidelines in 20 out of 20 trials. Nevertheless, removing of the bacteria from the HDCs has substantial drawbacks. Primary among these drawbacks is a profound increase in labor costs. Isolation of a typical dose (approximately 20 to 30 HDCs) takes an experienced technician between 10 and 20 min, and subsequent doses (isolated after the first) can take as little as 3 to 4 min each. In contrast, the separation of the HDCs from bacteria by hand requires approximately 60 to 120 min for each unit of 10 HDCs. Thus, manual separation of HDCs from bacteria may result in a 10-fold or more increase in labor costs. It is anticipated that future work may utilize new technology to mechanically separate HDCs from bacteria, and this technology may increase the clinical utility of HDCs. However, another disadvantage of separating the HDCs from bacteria via washing is that it is unknown whether or not extensive washing reduces the efficacy of the HDCs for therapeutic use. 

It is not evident that the separation of the HDCs from bacteria offers any particular advantage other than to meet regulatory guidelines in the US. Safety is apparently not a concern. Current commercial and community suppliers who do not store and ship HDCs for extended periods of time use HDCs in the raw form, without the removal of the associated bacteria from HDCs. These suppliers have found the associated bacteria to be benign, with no reported adverse reactions to bacteria even after tens of thousands of doses of HDCs have been consumed. The view that insect-associated bacteria are benign is further established by the fact that food poisoning from insect-associated bacteria is of no concern in any culture despite the extremely common occurrence of insects on Earth and the routine use of insects as a food source by humans in many cultures [40] and by other primates [41]. Further, insects are routinely consumed by Westerners in their food supply, and are recognized contaminants in a variety of food substances, including spices for which no cooking during food preparation is required. Insects are routinely consumed by Westerners in recreational or military “survival” situations. Such activity is openly supported by reputable sports authorities (e.g., Field and Stream Magazine [42]) and by military organizations, including the US Army [43].

Separation of HDCs from bacteria may hypothetically increase the shelf life of the organisms, but removing all of the bacteria in every preparation by mechanical washing may prove difficult. Because even a single bacterium may eventually lead to spoilage, a preservative may need to be added to HDC preparations for storage, even after removal of most bacteria. Importantly, the effects of various preservatives and storage times on the therapeutic effectiveness of HDCs are presently unknown, and the cost of determining this information adds a substantial burden to efforts aimed at making helminths available for widespread therapeutic use. 

Countries other than the US have thus far elected to regulate helminths as non-drug entities, and therefore major concerns regarding the impact of regulations on helminthic therapy as a drug may apply only to the US. We have argued [2] that the regulation of helminths as supplements or as other non-drug entities is potentially a major step forward in restoring the biota of humans living in Western cultures, a necessary step toward improved public health for people living in those cultures. 

The long-term goal of enriching the human biota with helminths is clear: We and others have concluded that all immunocompetent humans need regular exposure to helminths in order to maintain optimal immune function and avoid risk for inflammation-associated disease [7,8,44,45]. This conclusion is based on “biota alteration theory”, the view that loss of biodiversity from the ecosystem of the human body as a result of industrialization has contributed to increased immune dysregulation and non-adaptive inflammation [8]. In this view, exposure to helminths is a necessary component of our biology, and the essentially complete absence of those organisms is an underlying cause of inflammatory disease. Based on this view, we have argued that access to helminths is a basic human need and should be viewed as a basic human right, and that regulations involving use of helminthic therapy should take this into account [2].

It is expected that achieving the long-term goal of population-wide reintroduction of helminths will take place in phases. The first phase entails studies in humans to evaluate the potential for helminths to treat a wide range of inflammation-related diseases. The primary hurdle that has slowed progress in the past, the availability of a benign helminth that can be readily produced, is addressed in part by this work. In a second phase, we anticipate that commercial interests will eventually drive the use of helminths for widespread clinical use and for use by the general public, eventually as a supplement rather than a drug. It is expected that this advance will involve several specific developments:

(a) Regulatory hurdles in the US will need to be overcome, and benign helminths will become regulated as natural products or supplements rather than pharmaceuticals. This particular development is currently necessary only in the US and in other countries that utilize guidelines similar to those used by the FDA, as suggested above.
(b) Technology for the automatic isolation and packaging of helminths will be developed.
(c) Helminths other than the HDC will eventually come into use. Combinations of helminths may be used, and a wide range of helminths and even protozoans may prove useful [20]. Considerations involved in this evolution of helminth biotechnology will include effectiveness for treatment of disease, costs, and ease of delivery to the customer. At present, HDCs are not ideal in this regard because their short shelf life can complicate delivery to the customer.

As we have previously described [8,44,45], biota enrichment using helminths is a very promising field, but many unknowns exist. It remains unknown which organisms or combinations of organisms may work best for any disease, and the indications and contraindications for helminthic therapy are very poorly understood at present. It is hoped that the present study will provide a tool by which these unknowns can be probed in clinical trials. 

## Figures and Tables

**Table 1:** General effects of HDC exposure based on socio-medical studies.

Anticipated Effects of HDCs	Considerations/Caveats
Effective amelioration of autoimmune disease is often observed.	Based on observations made using helminths other than HDCs, it is expected that helminthic therapy works more frequently when the autoimmune disease is episodic, or waxes and wanes.
Allergic responses may be substantially retarded or even eliminated.	Reduction of allergic responses may be most effective in the absence of regular exposure to antigen.
Relief of neuropsychiatric conditions, including migraine headaches, anxiety disorders, chronic fatigue, and depression can be observed, even in cases where the condition has persisted for decades.	Therapy apparently does not eliminate dependency on pharmaceuticals that may have developed over time.
The effective dose and the maximum dose tolerated without adverse side effects are highly variable from individual to individual.	For a given individual, it is not yet possible to predict with certainty if therapy will be effective and what dose will be effective.
Both the minimum effective dose and the maximum dose tolerated without adverse side effects usually increases over time.	Periodic increases in exposure are generally needed and tolerated.
Individuals with mast cell dysfunction and with fibrotic disease (particularly fibromyalgia) may not respond well.	This is speculative and based primarily on the experience of self-treaters with helminths other than HDCs.
The most common adverse side effect is GI distress.	GI distress can generally be avoided by lowering the dose and increasing the frequency of administration if more therapeutic effect is desired.
In some pediatric patients, a common side effect is temporary hyperactivity, particularly when the organisms are first introduced or the dose is increased.	This side effect can be ignored, treated with ibuprofen, or avoided by lowered dose and increased frequency of administration.
In some pediatric patients, substantial adverse reactions may occur that include worsened behavior and severe GI distress. This reaction may be more than 20-fold less common than very positive reactions.	These reactions are apparently associated with colonization (adult HDs present in the GI tract), and require that the therapy be stopped and the HDs be removed by anti-helminth drugs. Fortunately, those drugs are effective.
Irritable bowel syndrome (IBS) and other digestive disorders, including IBD, can sometimes be relieved.	The degree of relief is apparently highly variable, depending on the individual.
Improved communication skills, learning ability and behaviors may be observed in some patients with autism.	This observation apparently applies to co-morbid inflammatory issues seen in some patients with autism. Effective treatment of impaired ability to understand social situations has not been observed.
Alleviation of a wide range of inflammation-associated conditions may be observed as a beneficial and unexpected side effect of attempting to treat an apparently unrelated condition. (e.g., hemorrhoids and cardiac arrhythmias have resolved in individuals attempting to treat allergies).	It is expected that alleviation of multiple inflammation-inducing factors (e.g., vitamin D deficiency, chronic psychological stress, inflammatory diets, cigarette smoking, etc.) will be synergistic with the beneficial effects of HDC exposure.
